# Analyzing Child Firearm Assault Injuries by Race and Ethnicity During the COVID-19 Pandemic in 4 Major US Cities

**DOI:** 10.1001/jamanetworkopen.2023.3125

**Published:** 2023-03-08

**Authors:** Jonathan Jay, Rachel Martin, Manish Patel, Kristal Xie, Faizah Shareef, Jessica T. Simes

**Affiliations:** 1 Department of Community Health Sciences, Boston University School of Public Health, Boston, Massachusetts; 2 Boston University Chobanian & Avedisian School of Medicine, Boston, Massachusetts; 3 Department of Sociology, Boston University College of Arts and Sciences, Boston, Massachusetts

## Abstract

This cross-sectional study examines changes in rates and disparities of fatal and nonfatal firearm assaults among children in New York, Los Angeles, Chicago, and Philadelphia before and during the COVID-19 pandemic.

## Introduction

Firearm violence in the United States has increased since the start of the COVID-19 pandemic, with particularly large increases in child injuries reported.^1,2^ Racial and ethnic disparities in all-age firearm injuries and deaths also appear to have grown.^3^ However, little research has examined how pandemic-related increases in firearm assaults may have disproportionally affected Black, Hispanic, and Asian children.

## Methods

In this cross-sectional study, we used data on firearm assaults (hereafter, shootings) with child (age <18 years) injuries from 2015 to 2021 in New York City, New York; Los Angeles, California; Chicago, Illinois; and Philadelphia, Pennsylvania. These represent the 3 most populous US cities, plus the city with more than 1 million population with the highest firearm homicide rate (Philadelphia). The Boston University institutional review board waived review and the requirement for informed consent because data were publicly available. We followed STROBE guidelines for cross-sectional research. Our data included both fatal and nonfatal shootings for each city except Chicago, where only fatal shootings were available for those younger than 18 years. Race and ethnicity was classified by police. We included Asian, Black, and White race and Hispanic and non-Hispanic ethnicity; other races appeared too infrequently for stable rate estimates. Using yearly population counts from the US Census, we calculated injury rates by racial and ethnic group and rates relative to the lowest-incidence group (ie, disparities). Rates for non-Hispanic Asian and non-Hispanic Black children were likely underestimates due to census data limitations (eMethods in Supplement 1). We treated March 15, 2020, as the pandemic start date.^4^

Next, we aggregated all racial and ethnic groups to estimate population-wide change associated with the pandemic. We used quasi-Poisson time series regression to model counts of child shootings for each week of the study period (ie, 365 weeks). The model included a linear term for year, a cubic B-spline with 7 equally spaced knots for week of year, a binary pandemic indicator, and a population offset. We ran the model separately for each city and ran a pooled model that included city fixed effects. We used a sandwich estimator to compute heteroskedasticity-robust SEs and estimated the number of pandemic-attributable injuries.

We used bootstrapping to generate confidence intervals for rates, disparities, and attributable counts. Analyses were conducted in R version 4.2.1 (R Project for Statistical Computing). We prespecified the level of significance as 2-sided 95% CIs.

## Results

Child shootings during the study period totaled 2672 (Table 1). The lowest rates (0.54 [95% CI, 0.40-0.68] per 100 000 person-years) were among non-Hispanic White children, whose rates did not increase during the pandemic. The highest rates were among non-Hispanic Black children (21.04 [95% CI, 20.11-21.99] per 100 000 person-years), whose rates increased. The Black-White disparity grew from a relative risk of 27.45 (95% CI, 21.03-36.22) before the pandemic to 100.66 (95% CI, 59.06-232.66) during the pandemic. Point estimates for Hispanic-White disparities tripled, and those for Asian-White disparities nearly tripled.

**Table 1.** Shooting Injuries Among Children Before and During the COVID-19 Pandemic, 2015 to 2021

Characteristic	Full study period (1/1/2015-12/31/2021)				Before pandemic (1/1/15-3/14/20)				During pandemic (3/15/20-12/31/21)			
	No.	Population, thousands (95% CI)^a^	Rate per 100 000 PY (95% CI)	RR (95% CI)	No.	Population, 1000s (95% CI)^a^	Rate per 100 000 PY (95% CI)	RR (95% CI)	No.	Population, 1000s (95% CI)^a^	Rate per 100 000 PY (95% CI)	RR (95% CI)
City												
New York, NY	703	1780 (1774-1787)	5.64 (5.22-6.07)	NA	422	1779 (1773-1785)	4.56 (4.13-4.99)	NA	281	1784 (1777-1792)	8.77 (7.74-9.80)	NA
Los Angeles, CA	693	827 (821, 834)	11.96 (11.12-12.84)	NA	418	837 (831-843)	9.60 (8.70-10.52)	NA	275	800 (793-808)	19.13 (16.98-21.36)	NA
Chicago, IL^b^	348	577 (572-583)	8.62 (7.72-9.53)	NA	251	584 (579- 590)	8.26 (7.24-9.28)	NA	97	556 (550-562)	9.71 (7.81-11.71)	NA
Philadelphia, PA	928	346 (343-349)	38.33 (35.89-40.81)	NA	539	346 (343-349)	29.98 (27.42-32.53)	NA	389	346 (342-351)	62.51 (56.41-68.78)	NA
Race and ethnicity (as exclusive categories)^c^												
Asian	38	556 (553-560)	0.98 (0.67-1.31)	1.81 (1.17-2.73)	24	546 (542-549)	0.85 (0.53-1.20)	1.39 (0.81-2.27)	14	588 (583-592)	1.33 (0.66-2.08)	3.94 (1.69-10.12)
Black	1899	1290 (1284-1295)	21.04 (20.11-21.99)	39.03 (30.68-52.52)	1130	1299 (1293-1304)	16.73 (15.78-17.72)	27.45 (21.03-38.22)	769	1263 (1256-1270)	33.90 (31.52-36.33)	100.66 (59.06-232.66)
Hispanic	679	1582 (1574-1589)	6.13 (5.68-6.59)	11.38 (8.89-15.45)	429	1575 (1567-1582)	5.24 (4.75-5.73)	8.60 (6.52-12.16)	250	1602 (1594-1611)	8.69 (7.64-9.76)	25.79 (14.80-60.60)
White	56	1484 (1479-1490)	0.54 (0.40-0.68)	1 [Reference]	47	1483 (1478-1488)	0.61 (0.44-0.79)	1 [Reference]	9	1488 (1481-1494)	0.34 (0.15-0.56)	1 [Reference]
Total	2672	3531 (3519-3542)	10.81 (10.42-11.21)	NA	1630	3546 (3535-3556)	8.84 (8.43-9.27)	NA	1042	3487 (3474-3500)	16.64 (15.65-17.63)	NA

Abbreviations: NA, not applicable; PY, person-years; RR, relative risk.

^a^ For population counts, 95% CIs are based on American Community Survey margins of error. Other 95% CIs in table are based on analyses of bootstrapped samples.

^b^ For Chicago, data set only included firearm homicides (ie, fatal shootings). All other cities include both fatal and nonfatal shootings.

^c^ Due to limitations in American Community Survey data, Asian and Black population counts include Hispanic population of the same race. For this reason, counts by race and ethnicity category sum to greater than total population count.

The pandemic was associated with nearly a 2-fold increase in child firearm assault rates (incidence rate ratio [IRR], 1.93; 95% CI, 1.65-2.29; *P* < .001) (Table 2). The estimated increase was largest in New York (IRR, 2.99; 95% CI, 2.09-4.28; *P* < .001). We estimated a pandemic-attributable increase of 503.5 child injuries across all cities (95% CI, 402.5-589.4 child injuries) from March 15, 2020, through December 31, 2021.

**Table 2.** Results of Regression Models Estimating COVID-19 Pandemic Effects on Child Firearm Assault Injuries^a^

Indicator	Population, thousands (95% CI)^b^	IRR (95% CI)	*P* value	Attributable No. (95% CI)
**Primary model (pooled)**				
COVID-19 indicator	34.87 (34.74 to 35.00)	1.93 (1.65 to 2.29)	<.001	503.4 (402.5-589.4)
**Secondary models (city-specific)**				
COVID-19 indicator				
New York City, NY	17.84 (17.77 to 17.92)	2.99 (2.09 to 4.28)	<.001	186.9 (144.8-227.3)
Los Angeles, CA	8.00 (7.93 to 8.08)	2.09 (1.51 to 2.90)	<.001	130.5 (95.6-188.9)
Chicago, IL^c^	5.56 (5.50 to 5.62)	1.23 (0.86 to 1.75)	.26	17.9 (−14.3 to 46.2)
Philadelphia, PA	3.46 (3.42 to 3.51)	1.51 (1.14 to 1.98)	.003	143.8 (62.3-193.1)

^a^ Attributable number refers to the estimated pandemic-attributable injuries, ie, the difference between total observed injuries and model-estimated total injuries in the counterfactual (ie, no COVID-19) scenario. Regression models were quasi-Poisson models with covariate controls for year (continuous) and week within year (as cubic B-spline with 7 equally spaced knots), with an offset for population count younger than 18 years (logged). (Covariates not shown.) Pandemic indicator was set to 1 for all weeks starting on or after March 15, 2020, and otherwise set to 0.

^b^ Population is based on counts reported during the pandemic period (see Table 1); 95% CIs are based on margins of error reported in American Community Survey data.

^c^ For Chicago, data set only includes fatal shooting injuries (firearm homicides).

## Discussion

In this study, child firearm assaults increased substantially during the COVID-19 pandemic in 4 major US cities. Racial and ethnic disparities increased, as Hispanic, Asian, and especially Black children experienced disproportionate shares of the increased violence.

One limitation of this study is that our design did not assess causes of these changes. However, our results are broadly consistent with research identifying sharper pandemic-associated violence increases in neighborhoods with less racial and economic privilege.^5^ Possible explanations include COVID-19’s exacerbation of inequities in access to health, employment, and educational resources.

The concentration of firearm victimization among Black, Hispanic, and Asian children must be addressed at the individual, community, and societal levels. It is critical to focus community safety and mental health interventions in the most affected communities and to target structural racism as a fundamental driver of the US firearm violence epidemic.

## Notes

Supplement 1.
eMethods.

eReferences.

Supplement 2.
Data Sharing Statement

## References

[1] Collings AT, Farazi M, Van Arendonk KJ, ; Midwest Pediatric Surgery Consortium. The COVID-19 pandemic and associated rise in pediatric firearm injuries: a multi-institutional study. J Pediatr Surg. 2022;57(7):1370-1376. doi:10.1016/j.jpedsurg.2022.03.034 35501165 PMC9001175

[2] Peña PA, Jena A. Child deaths by gun violence in the US during the COVID-19 pandemic. JAMA Netw Open. 2022;5(8):e2225339. doi:10.1001/jamanetworkopen.2022.25339 35925607 PMC9353595

[3] Pino EC, Gebo E, Dugan E, Jay J. Trends in violent penetrating injuries during the first year of the COVID-19 pandemic. JAMA Netw Open. 2022;5(2):e2145708. doi:10.1001/jamanetworkopen.2021.45708 35133435 PMC8826178

[4] Martin R, Rajan S, Shareef F, . Racial disparities in child exposure to firearm violence before and during COVID-19. Am J Prev Med. 2022;63(2):204-212. doi:10.1016/j.amepre.2022.02.007 35418336 PMC8921002

[5] Schleimer JP, Buggs SA, McCort CD, . Neighborhood racial and economic segregation and disparities in violence during the COVID-19 pandemic. Am J Public Health. 2022;112(1):144-153. doi:10.2105/AJPH.2021.306540 34882429 PMC8713621

