# Supplementary material for: Analyzing Child Firearm Assault Injuries by Race and Ethnicity During the COVID-19 Pandemic in 4 Major US Cities
Source: JAMA Netw Open. 2023 Mar 8;6(3):e233125. doi: 10.1001/jamanetworkopen.2023.3125 (PMC9996392; doi:10.1001/jamanetworkopen.2023.3125)
Supplement: Supplement 1. — eMethods. eReferences. [file jamanetwopen-e233125-s001.pdf]

Supplemental Online Content

Jay J, Martin R, Patel M, Xie K, Shareef F, Simes JT. Analyzing child firearm assault injuries by race and ethnicity during the COVID-19 pandemic in 4 major US cities. *JAMA Netw Open.* 2023;6(3):e233125. doi:10.1001/jamanetworkopen.2023.3125

eMethods.

eReferences.

This supplemental material has been provided by the authors to give readers additional information about their work.

© 2023 Jay J et al. *JAMA Network Open.*

eMethods.

Data

Shootings

We used 2015-2021 data on firearm assaults (“shootings”) with child injuries (age < 18) from New York City, Los Angeles, Chicago, and Philadelphia. January 1, 2015 was chosen as the start date because it was the earliest date for which data were available for all cities. Fatal and non-fatal shootings data were obtained from police department open data portals for NYC,[1] LA,[2] and Philadelphia.[3] LA and Philadelphia categorized injured persons by single-year age. NYC age categories included age < 18, consistent with the objectives of our study.

Chicago publishes similar shootings data,[4] but the youngest age category is under-20, which includes adults (ages 18-19). Including those individuals was not suitable for this study of child victimization, particularly because individuals ages 18-19 are expected to be overrepresented in the under-20 sample, since firearm victimization rates are higher at those ages. Therefore, we used Chicago data on age < 18 firearm homicide decedents (*i.e.*, fatal shootings only) obtained from the county medical examiner, which lists age by single year category.[5]

For each city, police assigned race/ethnicity for each injured person. Race and Hispanic ethnicity were categorized separately, such that we could (and did) generate mutually exclusive race/ethnicity categories, *i.e.,* Hispanic, non-Hispanic Asian, non-Hispanic Black, and non-Hispanic White. We did not include any other racial categories (*e.g.,* Native American, Multiracial) because these categories did not appear at sufficient rates in the data to generate stable estimates, and because it was not clear that these data were collected and reported systematically, based on their low rates of appearance in the data.

Population counts

To account for differences in child population, we obtained under-18 population counts from the American Community Survey (ACS) for each 5-year period ending 2015-2021. These data were obtained at the geographical level of “place,” which corresponded to the administrative boundaries of each city. Under-18 population counts were available for the full population (all races/ethnicities) and for our Hispanic and non-Hispanic White subgroups.

For our Asian and Black subgroups, the ACS only provides population counts that include both Hispanic and non-Hispanic individuals. Consequently, our firearm injury rate estimates for these subgroups include only non-Hispanic individuals in the numerator (shootings), but both Hispanic and non-Hispanic individuals in the denominator (population). We chose this approach because it likely generates conservative rate estimates compared to rates for the non-Hispanic White reference group. This decision mirrors recent work on Black and Hispanic youth firearm injury rates that used similar datasets.[6] On the other hand, the inflation of the denominator caused by inclusion of Hispanic individuals could be partially offset by the possible inclusion in the numerator of individuals who would have identified as another racial category on the Census (*e.g.,* Multiracial, Pacific Islander, Native American, “Other”) but were classified incorrectly by police.

We followed standard conventions to generate confidence intervals for injury rates. We divided ACS margins of error by 1.645 to generate standard errors. To aggregate standard errors across subcategories, we took the square root of the sum of squares.[6] A small number of entries for Hispanic child counts (N = 13 city-year entries) were missing margins of error in the American Community Survey data. Conservatively, we imputed these data by assuming that uncertainty in standard errors (SEs) for Hispanic children matched the greatest level of

© 2023 Jay J et al. *JAMA Network Open.*

uncertainty (*i.e.,* SEs as a share of population) found for any other group in the same city and year.

All data were publicly available and the Boston University Medical IRB waived review as non-human subjects research. To facilitate replication and additional research, the firearm injury data used in this study are also available at https://sites.bu.edu/riselab/.

Analysis

We hypothesized that the pandemic was associated with increased child victimization rates and that racial/ethnic disparities in child victimization increased across the study cities.

Descriptive analyses

We treated March 15, 2020, as the pandemic start date, consistent with prior research.[7] This date represents the first Monday after a presidential emergency declaration was announced on Saturday, March 13, 2020. While COVID transmission preceded this announcement, prior research has found that the third week of March 2020 was when behavioral patterns (*i.e.,* mobility) changed noticeably.[8]

We calculated firearm injury rates and racial/ethnic disparities for each city and for each racial/ethnic category, as described in the main text. Confidence intervals for population counts used standard errors calculated from Census margins of error, as described above. To calculate confidence intervals for firearm injury rates and disparities, we used bootstrapping. We conducted 10,000 iterations in which we sampled shootings from the original dataset, with replacement, to generate outcomes. We used these synthetic outcomes to calculate rates and disparities and report the 5th and 95th percentiles as the 95% CI in **Table 1**.

Time series analyses

We fitted quasi-Poisson time series regression models to weekly counts of child shootings by city, without disaggregating by race/ethnicity. Quasi-Poisson models were chosen because outcomes were overdispersed counts. Our models were designed to account for overall time trends, seasonal trends, population variation, and a level change associated with the pandemic. Each model included a linear term to capture long-term trends, a cubic B-spline for week of year to model seasonality, a binary indicator to capture COVID effects, and a logged population offset. Quasi-Akaike Information Criterion was used to identify the optimal number of knots in the time spline. The final model included 7 evenly-spaced knots. This approach to controlling for long-term trends and seasonality is consistent with recent approaches in time series epidemiology (e.g.,[9–11]). Our main (pooled) model included a city-level fixed effect. We also ran the model separately for each city without the fixed effect. We used a sandwich estimator to compute heteroskedasticity-robust standard errors.

Next, we used these models to estimate the change in injuries attributable to the pandemic. Conceptually, attributable number was the number of shootings that occurred, above and beyond the number that would have occurred if not for the COVID-19 pandemic. We calculated the counterfactual scenario by using each model to predict the total number of shootings when the COVID-19 indicator was artificially set to 0 for all time periods. We subtracted this predicted count from the count of actual shootings, such that pandemic-attributable number equaled shootings in the observed scenario (with pandemic) minus shootings in the counterfactual scenario (no pandemic). To generate confidence intervals for this attributable number, we used the same bootstrapping procedure described above, ran the pooled and city-specific models on each bootstrapped sample, and calculated the 5th and 95th percentiles for attributable number.

<u>Analytical considerations</u>. Since the goals of this analysis were descriptive rather than explanatory, we conceptualized pandemic effects solely as a level change. This approach

© 2023 Jay J et al. *JAMA Network Open.*

averaged over possible variation in the pandemic's effects over time, *e.g.,* widespread stay-at-home orders, re-openings, subsequent waves of infections, etc., because these sources of variation were not the focus of this research letter. Future work should examine possible slope changes, non-linearities, inflection points, etc., to improve understanding of violence dynamics during the pandemic period.

© 2023 Jay J et al. *JAMA Network Open.*

eReferences

1. NYPD Shooting Incident Data (Year To Date) | NYC Open Data. Accessed November 2, 2022. https://data.cityofnewyork.us/Public-Safety/NYPD-Shooting-Incident-Data-Year-To-Date-/5ucz-vwe8
2. Crime Data from 2020 to Present | Los Angeles - Open Data Portal. Accessed November 2, 2022. https://data.lacity.org/Public-Safety/Crime-Data-from-2020-to-Present/2nrs-mtv8
3. Shooting Victims - Datasets - OpenDataPhilly. Accessed November 2, 2022. https://www.opendataphilly.org/dataset/shooting-victims
4. Violence Reduction - Victims of Homicides and Non-Fatal Shootings | City of Chicago | Data Portal. Accessed November 2, 2022. https://data.cityofchicago.org/Public-Safety/Violence-Reduction-Victims-of-Homicides-and-Non-Fa/gumc-mgzr
5. Medical Examiner Case Archive | Cook County Open Data. Accessed November 2, 2022. https://datacatalog.cookcountyil.gov/Public-Safety/Medical-Examiner-Case-Archive/cjeq-bs86
6. Martin R, Rajan S, Shareef F, et al. Racial Disparities in Child Exposure to Firearm Violence Before and During COVID-19. *Am J Prev Med*. 2022;0(0). doi:10.1016/J.AMEPRE.2022.02.007
7. Jay J, Bor J, Nsoesie EO, et al. Neighbourhood income and physical distancing during the COVID-19 pandemic in the United States. *Nat Hum Behav*. 2020;4(December):1-28. doi:10.1038/s41562-020-00998-2
8. Bhaskaran K, Gasparrini A, Hajat S, Smeeth L, Armstrong B. Time series regression studies in environmental epidemiology. *Int J Epidemiol*. 2013;42(4):1187-1195. doi:10.1093/ije/dyt092
9. Gasparrini A, Armstrong B, Kenward MG. Distributed lag non-linear models. *Stat Med*. 2010;29(21):2224-2234. doi:10.1002/sim.3940
10. Bernal JL, Cummins S, Gasparrini A. Interrupted time series regression for the evaluation of public health interventions: A tutorial. *Int J Epidemiol*. 2017;46(1):348-355. doi:10.1093/ije/dyw098

© 2023 Jay J et al. *JAMA Network Open.*
