# Supplementary material for: Analyzing Child Firearm Assault Injuries by Race and Ethnicity During the COVID-19 Pandemic in 4 Major US Cities
Source: JAMA Netw Open. 2023 Mar 8;6(3):e233125. doi: 10.1001/jamanetworkopen.2023.3125 (PMC9996392; doi:10.1001/jamanetworkopen.2023.3125)
Supplement: Supplement 2. — Data Sharing Statement [file jamanetwopen-e233125-s002.pdf]

Data Sharing Statement

Jay. Analyzing Child Firearm Assault Injuries by Race and Ethnicity During the COVID-19 Pandemic in 4 Major US Cities. *JAMA Netw Open*. Published March 08, 2023. doi:10.1001/jamanetworkopen.2023.3125

Data
Data available: Yes
Data types: Data (not involving human participants)
How to access data: sites.bu.edu/riselab
When available: With publication

Supporting Documents
Document types: Statistical/analytic code
How to access documents: sites.bu.edu/riselab
When available: With publication

Additional Information
Who can access the data: Fully open source
Types of analyses: Any purpose
Mechanisms of data availability: No permissions required
Any additional restrictions: Data are already publicly available, from different sources; for greater convenience, we will provide the cleaned data for download in a centralized location.
